# Distributed Acoustic Sensing Using Chirped-Pulse Phase-Sensitive OTDR Technology

**DOI:** 10.3390/s19204368

**Published:** 2019-10-09

**Authors:** María R. Fernández-Ruiz, Luis Costa, Hugo F. Martins

**Affiliations:** 1 Department of Electronics, University of Alcalá, Alcalá de Henares, 28805 Madrid, Spain; rosario.fernandezr@uah.es (M.R.F.-R.); luis.duarte@uah.es (L.C.); 2 Instituto de Óptica, CSIC, 28006 Madrid, Spain

**Keywords:** distributed acoustic sensing, Rayleigh scattering, optical time-domain reflectometry, chirped-pulse, phase-sensitive OTDR

## Abstract

In 2016, a novel interrogation technique for phase-sensitive (Φ)OTDR was mathematically formalized and experimentally demonstrated, based on the use of a chirped-pulse as a probe, in an otherwise direct-detection-based standard setup: chirped-pulse (CP-)ΦOTDR. Despite its short lifetime, this methodology has now become a reference for distributed acoustic sensing (DAS) due to its valuable advantages with respect to conventional (i.e., coherent-detection or frequency sweeping-based) interrogation strategies. Presenting intrinsic immunity to fading points and using direct detection, CP-ΦOTDR presents reliable high sensitivity measurements while keeping the cost and complexity of the setup bounded. Numerous technique analyses and contributions to study/improve its performance have been recently published, leading to a solid, highly competitive and extraordinarily simple method for distributed fibre sensing. The interesting sensing features achieved in these last years CP-ΦOTDR have motivated the use of this technology in diverse applications, such as seismology or civil engineering (monitoring of pipelines, train rails, etc.). Besides, new areas of application of this distributed sensor have been explored, based on distributed chemical (refractive index) and temperature-based transducer sensors. In this review, the principle of operation of CP-ΦOTDR is revisited, highlighting the particular performance characteristics of the technique and offering a comparison with alternative distributed sensing methods (with focus on coherent-detection-based ΦOTDR). The sensor is also characterized for operation in up to 100 km with a low cost-setup, showing performances close to the attainable limits for a given set of signal parameters [≈tens-hundreds of pe/sqrt(Hz)]. The areas of application of this sensing technology employed so far are briefly outlined in order to frame the technology.

## 1. Introduction

Sensing is a fundamental tool that provides information about the characteristics of the surrounding environment to electronic systems. This information can be collected for analytical purposes or processed and employed to take specific actions. Sensors have become ubiquitous nowadays, as they are present in most of our daily used items. A particularly interesting example are distributed optical fibre sensors, which employ light to probe a kilometer-length optical fibre used as the sensing element. Within the spatial resolution, typically in the meter scale, distributed optical sensors can determine strain or temperature variations along the fibre length. The light that interrogates the fibre simultaneously carries the perturbation information to a receiver, enabling immediacy in the perturbation detection with a very simple and cost-effective setup.

Distributed optical fibre sensors rely on scattering processes occurring along the fibre, either Rayleigh, stimulated Brillouin or Raman scattering. Sensors based on each of these scattering processes have distinctive features that make them more or less suitable for different applications. For example, Raman-based sensors are very effective temperature sensors [1], those based on Brillouin can interrogate very long fibres resolving up to a million points (cm resolution) [2], while Rayleigh-based ones can measure dynamic variations up to the MHz regime (acoustic frequencies) [3]. Remarkable development has been made in the last decade to achieve better resolution, higher bandwidth or longer range operation using these technologies. In the literature, it is possible to find excellent reviews about the state of the art of the different types of distributed optical fibre sensors [4,5,6,7,8,9]. A few years ago, a novel methodology to interrogate an optical fibre using Rayleigh backscattering was proposed and demonstrated. This technique relies on phase-sensitive optical time-domain reflectometry (ΦOTDR) technology using direct detection, but instead of using transform-limited pulses (as it is the general case), it uses a train of linearly-chirped optical pulses. The technique was therefore named chirped-pulse (CP-)ΦOTDR [10]. This simple alteration of the conventional setup substantially alters the way a perturbation on the fibre is detected and quantified, greatly simplifying the traditionally used methodology. Besides, derived from its principle of operation, CP-ΦOTDR presents an extraordinary robustness against laser phase noise and a record measurand sensitivity [11,12].

This paper reviews the basic theoretical concepts and principle of operation underlying this novel distributed sensing procedure. The typical experimental setup is shown and the sensing features of this technique are analyzed in detail and compared with those from traditional ΦOTDR configurations. Finally, the use of CP-ΦOTDR in several classical applications of distributed fibre optics sensing are discussed, together with novel sensing and processing applications of the technique.

## 2. Principle of Operation of CP-ΦOTDR

Similar to conventional OTDR, ΦOTDR schemes interrogate an optical fibre through the propagation of a train of typically rectangular-like optical pulses and subsequent analysis of the Rayleigh backscattered light. In the phase-sensitive method however, the propagating light is coherent, in such a way that the light reflected by each scattering center (i.e., defect, molecule or ion intrusion, or in general any inhomogeneity in the fibre) adds up coherently upon reception forming a speckle-like pattern. The backscattered pattern remains unaltered until some external perturbation (e.g., temperature or strain) locally changes the phase relationship between light scattered from local reflectors. As a result, there is a linear relation between the change in accumulated phase and the induced perturbation. After direct photodetection, however, this translates into a non-linear local change in the amplitude of the recovered power trace. If the trace is coherently detected, the perturbation can be quantified by using the differential trace phase [13,14]. Alternatively, perturbations can also be quantified using direct detection by using the restorability principle of Rayleigh backscatter traces: a local change of refractive index of the fibre at a particular position can be compensated by a frequency shift of the launched pulse, which is proportional to the optical path variation (i.e., proportional to the perturbation magnitude). This effect has been exploited to quantify the perturbation by sweeping the frequency of the probe pulses launched into the fibre and searching trace-to-trace for the particular frequency shift that compensates the effect of the perturbation over a particular time window [15]. Typically, the optical circuits required in the schemes enabling perturbation quantification (i.e., those using coherent-detection or probe frequency sweeping) are more complex and demand higher system stability and higher performance of the laser source, increasing the sensor cost.

In 2016, an original ΦOTDR interrogation method was introduced and tested, giving rise to a sensing technique known as chirped-pulsed (CP-)ΦOTDR [10]. This method relies on the same principle of traditional ΦOTDR using direct detection. The fundamental difference is that the probe pulses have a linear chirp, i.e., a linear variation of the instantaneous frequency along the pulse width, instead of being transform-limited pulses as in the traditional case.

### 2.1. Mathematical Description

The probe pulse can be analytically modeled as $e_{p}\left(t\right)={\hat{e}}_{p}\left(t\right)\exp\left\{j2\pi \upsilon_{0}t\right\}$, where $\upsilon_{0}$ is the pulse central frequency and the complex amplitude ${\hat{e}}_{p}\left(t\right)$ is:

(1) $${\hat{e}}_{p}\left(t\right)=\mathrm{rect}\;\left\{\frac{t}{\tau_{p}}\right\}\cdot \exp\left\{-j2\pi \frac{\delta \upsilon_{p}}{2\tau_{p}}t^{2}\right\}=\hat{a}\left(t\right)\cdot \exp\left\{-j2\pi \frac{\delta \upsilon_{p}}{2\tau_{p}}t^{2}\right\}.$$

In Equation (1), rect{·} is the rectangular function, with $\tau_{p}$ being the pulse width, and $\delta \upsilon_{p}$ is the maximal excursion of the instantaneous frequency, corresponding to the chirp-induced spectral content. The spectrum of the probe pulse is calculated as the convolution of the Fourier transform of the two terms multiplied in Equation (1):

(2) $$\begin{array}{cc} {\hat{E}}_{p}\left(\omega \right) & =\sqrt{\frac{\tau_{p}}{\delta \upsilon_{p}}}\int_{-\infty }^{\infty }\hat{A}\left(\Omega \right)\cdot \exp\left\{j\frac{\tau_{p}}{4\pi \delta \upsilon_{p}}{\left(\omega -\Omega \right)}^{2}\right\}d\Omega \\ & =\sqrt{\frac{\tau_{p}}{\delta \upsilon_{p}}}\exp\left\{j\frac{\tau_{p}}{4\pi \delta \upsilon_{p}}\omega^{2}\right\}\cdot \int_{-\infty }^{\infty }\hat{A}\left(\Omega \right)\cdot \exp\left\{j\frac{\tau_{p}}{4\pi \delta \upsilon_{p}}\Omega^{2}\right\}\cdot \exp\left\{-j\frac{\tau_{p}}{2\pi \delta \upsilon_{p}}\omega \Omega \right\}d\Omega , \end{array}$$

where $\hat{A}\left(\omega \right)$ is the Fourier transform of $\hat{a}\left(t\right)$, ω=2πυ is the angular frequency and Ω is an auxiliary variable with units of angular frequency. If $\hat{A}\left(\omega \right)$ is confined to a well-defined spectral band $2\pi \Delta B_{A}$ and the chirp-induced spectral content $\delta \upsilon_{p}$ is sufficiently large that:

(3) $$\frac{\tau_{p}}{\delta \upsilon_{p}}\Delta {B_{A}}^{2}\ll 1,$$

the phase factor $\left(\tau_{p}\Omega^{2}/4\pi \delta \upsilon_{p}\right)\leq \left(\tau_{p}{\left(2\pi \Delta B_{A}\right)}^{2}/4\pi \delta \upsilon_{p}\right)$ is negligible, and Equation (2) can be approximated by:

(4) $$\begin{array}{cc} {\hat{E}}_{p}\left(\omega \right) & \propto \exp\left\{j\frac{\tau_{p}\omega^{2}}{4\pi \delta \upsilon_{p}}\right\}\cdot \int_{-\infty }^{\infty }\hat{A}\left(\Omega \right)\cdot \exp\left\{-j\frac{\tau_{p}}{2\pi \delta \upsilon_{p}}\omega \Omega \right\}d\Omega \\ & =\exp\left\{j\frac{\tau_{p}\omega^{2}}{4\pi \delta \upsilon_{p}}\right\}\cdot F^{-1}\left\{\hat{A}\left(\Omega \right)\right\}=\exp\left\{j\frac{\tau_{p}\omega^{2}}{4\pi \delta \upsilon_{p}}\right\}\cdot {\hat{a}\left(t\right)|}_{t=-\frac{\tau_{p}}{2\pi \delta \upsilon_{p}}\omega }, \end{array}$$

where F{⋅} denotes the Fourier transform. The condition in Equation (3) leads to a frequency-to-time mapping of the probe pulse spectral envelope, with the frequency-time conversion factor indicated in Equation (4). When using rectangular probe pulses $\Delta B_{A}∼1/\tau_{p}$, this condition simplifies to the fact that the chirp-induced spectral content must be much larger than the transform-limited pulse bandwidth. If this condition is satisfied, there is a linear relationship between the time-domain signal and its spectrum (plus a quadratic phase term), in such a way that any variation in one domain is reproduced in the other one. This frequency-to-time-mapping [16] produces that a perturbation-induced spectral shift Δυ in the trace maps into a local temporal delay Δt in the trace, following the relationship in the sub index of the right hand-side of Equation (4), namely:

(5) $$\Delta t=-\frac{\tau_{p}}{\delta \upsilon_{p}}\Delta \upsilon .$$

**Figure 1:**
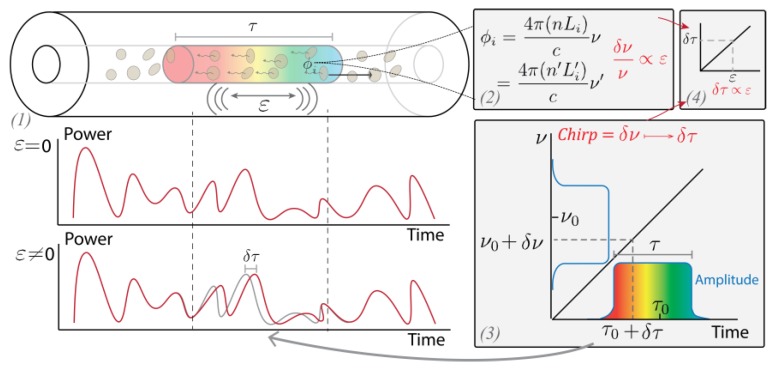
(From Ref. [12]) Working principle of chirped-pulse ΦOTDR: (**1**) A linearly chirped optical pulse propagates along the fibre, and a small fraction of the power is elastically backscattered. (**2**) The optical trace from the interference of the backscattered light will vary if a perturbation over the fibre locally altering the optical path distance (i.e., changes to the refractive index n or length L_i_) occurs. A frequency detuning may compensate for the change in phase, thus recovering the previous optical trace. (**3**) The pulse linear chirp maps this frequency detuning into a temporal delay within the pulse window, in such a way that the optical trace contains a local time delay proportional to the perturbation, @2019 IEEE [12].

This Δt can be related to ongoing perturbations of group refractive index variation ($\Delta n_{g}$), temperature (ΔT) or strain (Δε) by [10,15]:

(6) $$-0.78\cdot \Delta \varepsilon \approx -(6.92\cdot 10^{-6})\cdot \Delta T\approx \frac{\Delta n_{g}}{n_{g}}=\frac{\Delta \upsilon }{\upsilon_{0}}=-\frac{1}{\upsilon_{0}}\frac{\delta \upsilon_{p}}{\tau_{p}}\Delta t,$$

with $n_{g}$ being the group refractive index of the fibre. The relationship attained in Equation (6) was also obtained from an alternative derivation in [10], where the interference of the backscattering of the pulse along its propagation is analyzed for each instant at photodetection. The interested reader is invited to review both derivations for a better understanding of the operation principle of the technique. The temporal delay Δt is maintained in the photodectected intensity trace, so that simple direct detection of the backscattered light enables both detection and quantification of the perturbation. The perturbation-induced temporal shift is detected by trace-to-trace moving correlations, converting the fibre interrogation into a time-delay estimation (TDE) problem. The effect of the perturbation in an optical fibre interrogated with a CP-ΦOTDR scheme is depicted in Figure 1.

### 2.2. Typical Setup

**Figure 2:**
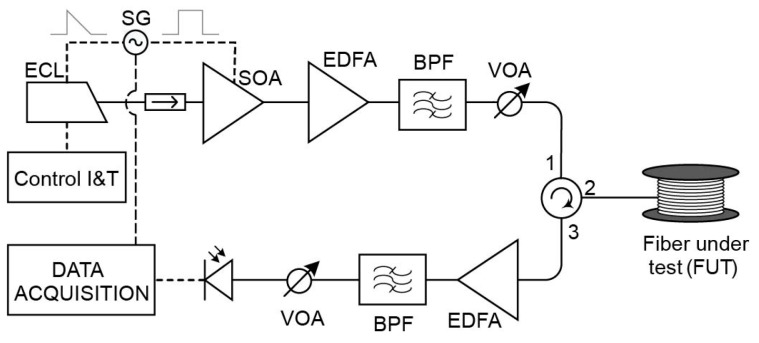
Setup of CP-ΦOTDR. ECL: External cavity laser; SG: Signal generator; I&T: Intensity and temperature; SOA: Semiconductor optical amplifier; EDFA: Erbium doped fibre amplifier; BPF: Band-pass filter; VOA: Variable optical attenuator; FUT: Fibre under test; PD: Photodetector. Solid line represents optical path, dashed line represents electrical path.

As described above, CP-ΦOTDR can be implemented using an optical setup nearly identical to that of a traditional ΦOTDR used for distributed vibration monitoring, i.e., using simply direct detection. The only modification required is that the probe pulse must include a sufficiently high linear chirp (accomplishing Equation (3)). To date, this linear chirp has been typically induced in the probe pulses by linearly modulating the current driver of a butterfly-package laser diode/external cavity laser (ECL) [10]. The final setup is shown in Figure 2.

The ECL emits a monochromatic continuous-wave (CW) light at frequency $\upsilon_{0}$. A semiconductor optical amplifier (SOA) is used to pulse the optical source. The SOA provides a high suppression of the intra-band coherent noise thus increasing the signal-to-noise ratio (SNR) of the received traces [17]. The electrical train of pulses used to drive the SOA are synchronized with an electrical periodic ramp signal that drives the current driver of the ECL. The ramp slope is designed so that the instantaneous frequency of the pulse changes $\delta \upsilon_{p}$ over the total pulse duration, inducing this way the target linear chirp. Alternatively, the chirped pulse probe can be generated by electro-optical modulation of the output of a CW laser. It should be noted that the use of direct laser current modulation to apply chirp requires a (single) initial calibration, since the dependency of the applied chirp VS applied current is not known a priori for an arbitrary laser. However, it has the advantage of providing a simpler/cheaper method to apply the intended chirp modulation, when compared to the use of external electro-optical modulation. Additionally, the use of direct laser current modulation does not impose optical losses to the signal (as is typically the case with external electro-optical modulation). Therefore, to date, this has been the preferred method. From this point on, the circuit is identical to a traditional direct-detection ΦOTDR scheme. After an optical isolator, there is an amplification stage composed of an erbium-doped fibre amplifier (EDFA) and a band pass filter (BPF) aimed at reducing amplifier spontaneous emission (ASE). The resulting probe is then launched into the fibre under test (FUT) through an optical circulator. The backscattered light received at the launching end of the fibre is subsequently amplified via another amplification stage (EDFA + BPF). The resulting signal is then photodetected and electrically recorded.

### 2.3. Basic Measurement Settings

In general, the operation principle and measurands of CP-ΦOTDR differ from those of a traditional direct-detection-based (nonlinear) DAS. However, the basic measurement settings of CP-ΦOTDR, namely, the spatial resolution, the acoustic sampling and the sensing range, present similar characteristics as any OTDR-based distributed sensor, since those are related to the pulsed operation.

#### 2.3.1. Acoustic Sampling

To avoid overlapping in detection of optical traces generated by subsequent pulses, the acoustic sampling $f_{s,ac}$ (pulse repetition rate) is limited by the time of flight of the pulses in the fibre:

(7) $$f_{s,ac}=\frac{c}{2n_{g}L},$$

where c is the speed of light in vacuum and L is the fibre length. Hence, the maximum readable frequency is limited by the Nyquist theorem to $f_{s,ac}/2$.

#### 2.3.2. Spatial Resolution and Gauge Length

The “optical” spatial resolution is defined as the length of the optical trace that shows variations when applying a punctual (spatially) strain perturbation to the fibre. In other words, it is the minimum distance between two strain perturbations applied to the fibre for them to cause independently resolved variations in the optical fibre trace. Just as any OTDR-based sensor, the optical spatial resolution is set by $c\cdot \tau_{p}/\left(2n_{g}\right)$ (e.g., $\tau_{p}$ = 100 ns will set an optical spatial resolution of ≈10 m).

Similar to a traditional OTDR, the pulse energy needs to be large enough to ensure a usable trace SNR. The use of shorter pulses (higher spatial resolution) will generate optical traces with lower optical powers, and therefore reduce the sensing range and/or measurement sensitivity. In CP-ΦOTDR however, a second condition exists regarding the pulse width: the $\delta \upsilon_{p}$ must be much larger than the transform-limited pulse bandwidth (Equation (3)). This makes operation with sub-meter spatial resolution unpractical (although feasible), as the required $\delta \upsilon_{p}$ is increased (typically to several GHz). The use of sub-band processing has been recently proposed to allow increasing the spatial resolution beyond $c\cdot \tau_{p}/\left(2n_{g}\right)$ in CP-ΦOTDR but further study is required to consolidate the technique [18].

Regarding the “measurand” spatial resolution, it is defined for CP-ΦOTDR as the minimum distance between two punctual (spatially) strain perturbations applied to the fibre for them to be independently measured, taking into account the required TDE processing. This is set by the convolution between the “optical” spatial resolution and the window used to compute the time delays (cross-correlation time window, $\tau_{corr}$). The parameter $\tau_{corr}$ finds its parallelism in the “gauge length” defined for coherent-detection ΦOTDR (distance used to compute phase-difference between two fibre points, in order to compute the strain applied between those two points; typically larger than the spatial resolution).

In practice however, in CP-ΦOTDR $\tau_{corr}$ is typically set to $\tau_{p}$, thus optimizing the “measurand” spatial resolution, which (assuming a square-like optical pulse) results in a “measurand” impulse response which presents a spatial FWHM of the same size of the “optical” resolution. In this case:

(8) $$‘\mathrm{Optical}’\;\mathrm{spatial}\;\mathrm{resolution}\;=\;‘\mathrm{Measurand}’\;\mathrm{spatial}\;\mathrm{resolution}\;=\;c\cdot \tau_{p}/\left(2n_{g}\right).$$

#### 2.3.3. Sensing Range

This parameter is defined as the maximum length in which measurements are reliable, and it is intimately related to the optical trace SNR. The use of TDE-based measurement in CP-ΦOTDR shows a high tolerance to optical noise, allowing for proper operation with optical trace SNRs close to 0 dB (e.g., operation down to ≈1.5 dB optical SNR (≈3 dB electric SNR) is discussed in Section 5), while maintaining tens-hundreds pε/√Hz sensitivities. The sensing range depends on several parameters, such as measurement settings, performance of the used components on the optical setup, chirp content, etc. However, measurements ranges of 30–50 km and 80–100 km with distributed optical amplification are typically attainable, even for a low complexity/cost setup (see Section 5).

### 2.4. Detection Bandwidth Considerations

An increased $\delta \upsilon_{p}$ requires higher detection bandwidth, leading to higher noise in the detection process. At first glance, this may seem as a limiting factor in the measurable strain sensitivity and/or sensing range. However, increasing the $\delta \upsilon_{p}$ (and therefore the fibre trace spectral content) also increases both the time-bandwidth product (Equation (3)) for the correlation, as well as the system tolerance to static optical SNR. In fact, while increasing the detection bandwidth decreases the SNR of the fibre trace (considering additive white Gaussian noise in detection), a higher $\delta \upsilon_{p}$ improves the performance of the strain estimation [12] (refer to Section 4.2 for a full discussion between acoustic and electrical SNR, and signal bandwidth). In addition, the increased bandwidth increases the robustness of the TDE process, thus leading to an increase in measurable distance [19,20]. Overall, it can be concluded that, although counterintuitive, the use of higher pulse chirp ($\delta \upsilon_{p}$) (and consequently higher detection bandwidth) shall not decrease the system performance, and even certain improvement may be expected.

### 2.5. Type of Measurement: Local Measurement (vs. Spatially Differentiated)

In CP-ΦOTDR, the local strain variation applied to the fibre (accumulated between two temporal instants) is directly measured in each spatially resolved point. This capability is related to the use of direct detection, which transduces a local fibre perturbation into a local disturbance of the optical trace. This means that even if the entire fibre length is simultaneously perturbed, the system is able to discriminate the strain applied in each spatially resolved point, with (almost) no cross-talk with neighboring points.

Residual cross-talk can occur due to (i) accumulated polarization changes and/or (ii) accumulated flight time delay in propagating optical pulse due to fibre optical path changes. While a full discussion on these issues is yet to be addressed, these effects are usually too small to have a relevant impact in the system. E.g., for the case of (ii), for an average temperature shift of ~12 K accumulated along 16.6 km, a “virtual” temperature shift of ~80 mK was measured, i.e., a residual cross-talk coefficient of approximately ~5 × 10^−7^ K/(K·m), under typical working conditions ($\tau_{p}$ = 100 ns, $\delta \upsilon_{p}$ = 1 GHz) [21]. In any case, since the accumulated perturbations leading to a certain fibre position can be measured, this effect can be fully compensated in post-processing.

In the case of coherent-detection ΦOTDR however, the perturbations occurring along the fibre are transduced into an accumulated (and not local) phase-shift of the trace. The measurement is therefore required to be spatially differentiated, i.e., the phase shift is computed between two positions of the fibre separated by a certain distance (gauge length), in order to determine the strain occurring between those two points (otherwise any perturbation in a point resulting in >2π phase-shift would impair the measurements in all subsequent fibre points).

While this procedure is ultimately similar to the local measurement of CP-ΦOTDR, in coherent-detection ΦOTDR the strain sensitivity/dynamic range and strain spatial resolution (length over which strain can be measured and discriminated from strain perturbations applied in neighboring points without the existence of cross-talk) is associated with the gauge length rather than directly with the spatial resolution, typically defined by ½ the pulse width.

## 3. Pulse Propagation and Optical Trace

When computing pulse propagation dynamics, the typical $\delta \upsilon_{p}$ (≤ few GHz) is too small to have a relevant impact (in terms of nonlinearities, dispersion and distributed amplification). Note that the dynamics concerning Brillouin effect will change significantly when the $\delta \upsilon_{p}$ is larger than the Brillouin gain bandwidth, but this effect is not relevant in typical ΦOTDR operation.

Therefore, as a first approximation, the physics describing pulse propagation in CP-ΦOTDR resemble those of traditional ΦOTDR with transform-limited pulses. In this section, an overview on the pulse propagation and optical trace of ΦOTDR is presented, comparing dynamics of both transform-limited-pulse (classical) and chirped-pulse-based implementations.

### 3.1. Dispersion

Considering an example of a typical CP-ΦOTDR probe pulse, with $\tau_{p}$ = 100 ns and $\delta \upsilon_{p}$ = 1 GHz (≈0.008 nm), propagating along a conventional single-mode fibre (SMF), with a dispersion of ≈18 ps/(nm·km) [22], then a 1% pulse temporal broadening (1 ns) is expected only after 7000 km, which largely exceeds typical operation ranges, even if optical repeaters are considered. Therefore, in the linear regime, dispersion should have a negligible impact in the performance of these systems, due to the relatively small pulse spectral contents.

### 3.2. Nonlinearities—Modulation Instability

In distributed optical sensors, the sensing range, spatial resolution, and measurement SNR are tightly related parameters. By increasing the input pulse peak power, the optical SNR and sensing range can be increased without sacrificing spatial resolution, but the maximum usable pulse peak power is limited by the onset of nonlinear effects. For typical ΦOTDR operation ($\tau_{p}$ = 10−100 ns, over fibre lengths of few km to 100 km), the input pulse peak power is limited by the occurrence of modulation instability (MI) [23].

**Figure 3:**
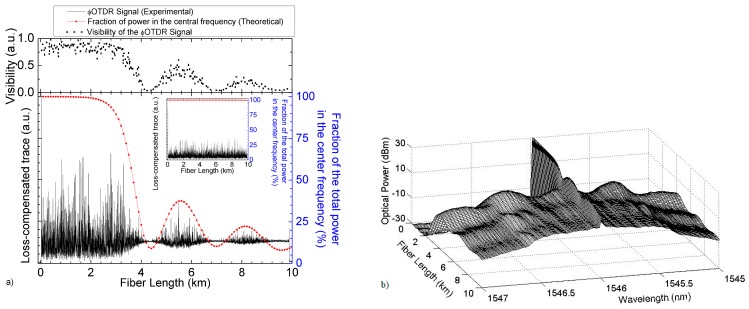
(From [23]) (**a**) ΦOTDR signal along the fibre under test for an input pump peak power of ~1.25 W (main figure) and ~0.35 W (inset figure). Fibre losses have been eliminated along the trace to improve visualization. The theoretical fraction of power contained in the central wavelength is also presented in both cases. The top figure shows the visibility of the ΦOTDR interference signal for the main figure signal. The visibility is computed as V = (T_max_ − T_min_)/(T_max_ + T_min_), where T_max_ and T_min_ are the maximum and minimum values of the trace over a certain distance record (in this case, a window of 40 m); (**b**) Simulation of the input pulse spectrum evolution along the fibre using the parameters used in (**a**). @2013 The Optical Society [23].

In optical fibres, MI arises from a combination of the Kerr effect and anomalous dispersion during pulse propagation. This results in the build-up of two frequency sidebands, typically separated by tens-hundreds of GHz [24], symmetrically placed around the pulse central optical frequency. This leads to a decrease of coherence of the pulse propagating (and a decrease of the pulse power in its central frequency), causing a decrease of the visibility of the optical trace backscattered and a decrease of the measurable strain SNR. A reversible power exchange between the pump and the sidebands known as the Fermi–Pasta–Ulam (FPU) recurrence [25] will occur during propagation in the strong conversion regime. The dynamics of MI are illustrated in Figure 3a,b (see caption for details).

The input pulse peak power threshold for the onset of MI (P_MI_) to be generated over long SMF (> effective fibre length, i.e., >~20 km), is typically ~200 mW [23,26], depending on the used pulse shapes and fibre parameters). However, with the use of Raman amplification, the pulse peak is maintained at higher powers over longer fibre lengths, and therefore the P_MI_ will be significantly lower.

**Figure 4:**
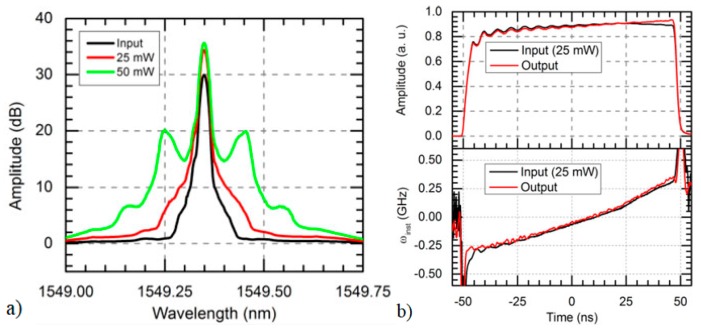
(From [27]) Chirped pulse characteristics after propagation along 75 km of SMF using bidirectional first-order Raman amplification, for different (25 mW, 50 mW) input pulse peak powers. The reference pulse is represented in black. (**a**) Measured optical spectrum. (**b**) Amplitude and chirp profile, @2017 IEEE [27].

Figure 4 (from [27]) shows the optical spectrum of chirped pulses with different input peak powers, after propagation over 75 km with bidirectional first-order Raman amplification. In this case, MI sidebands at the end of the fibre start to be noticeable for a pulse peak power of 50 mW.

#### Mitigation of MI in ΦOTDR

**Figure 5:**
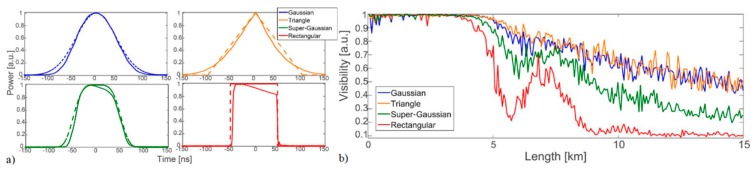
(From [26]) (**a**) Optical pulses with different intensity profiles (similar FWHM) used to probe a ΦOTDR system and (**b**) Correspondent ΦOTDR trace visibility for each pulse (all with the same pulse energy of 165 nJ), showing a clear performance improvement from square to other intensity profiles. @2016 The Optical Society [26].

It has been demonstrated that the impact of MI can be mitigated by acting on the pulse intensity profile [26]. The use of squared pulses has been proven to be detrimental, while the use of smoother profiles, such as Gaussian or super-Gaussian will decrease the impact of MI on the ΦOTDR performance. Figure 5 overviews the impact of MI on the ΦOTDR trace visibility for different pulse intensity shapes (see caption for details).

**Figure 6:**
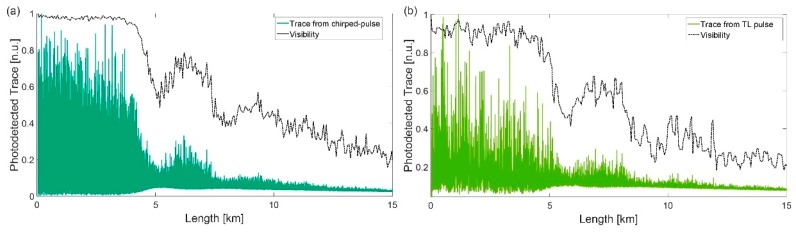
Comparison of the impact of MI in the optical traces for (**a**) for chirped pulse (using the experimental setup described in Section 2.2, with $\delta \upsilon_{p}$ ≈ 400 MHz); (**b**) transform-limited (TL) pulse (@2016 The Optical Society [26]), using similar pulse intensity shapes (super-Gaussian, ≈100 ns FWHM) and pulse energies (165 nJ per pulse).

Regarding the impact of MI in CP-ΦOTDR, Figure 6 shows the optical traces obtained using transform-limited pulses and chirped pulses, employing similar pulse intensity shapes and peak powers. The comparison demonstrates that MI leads to a similar visibility impact along the fibre (in terms of starting point, secondary lobs and overall amplitude along the fibre) in both cases.

No in-depth study has been performed on the impact of MI when using large $\delta \upsilon_{p}$ (i.e., ≥ tens of GHz, when $\delta \upsilon_{p}$ is comparable to the MI sidebands frequency gain bandwidth and separation). However, for typical CP-ΦOTDR operation, where $\delta \upsilon_{p}$ lower than few GHz are employed, the impact of MI, and strategies to mitigate its effect, turn out to resemble those of traditional transform-limited ΦOTDR.

### 3.3. Distributed Optical Amplification

In this section, the use of Raman amplification to extend the measurable range of CP-ΦOTDR is discussed. Different implementations of distributed optical amplification have been successfully used to increase the optical SNR along the fibre [27,28,29,30,31,32], and therefore extend the measurable range of distributed optical fibre sensors. Alternatively, optical repeaters can be used [33]. However, depending on the application, this may present a practical problem due to the requirement of energy supply in the middle of the sensing fibre.

While the Brillouin effect is not relevant in the majority of ΦOTDR schemes, its use to provide distributed optical amplification in ΦOTDR has been demonstrated under specific conditions [28] (note that the Brillouin frequency shift (BFS) is dependent on the fibre temperature and BFS detuning was required along the fibre). However, the use of Brillouin effect for distributed optical amplification of chirped pulses requires significant implementation changes (the typical Brillouin gain bandwidth—~50 MHz for SMF—is lower than the spectral content of chirped pulses and larger than the spectral content of transform-limited pulses under normal operation), and such discussion is out of the scope of this paper.

**Figure 7:**
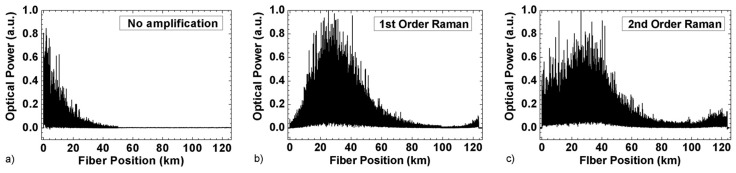
(From [32]) ΦOTDR (using transform-limited pulses) optical trace: (**a**) Without Raman amplification (obtained using the experimental setup used in [29], but without Raman amplification and 200 mW input peak power); (**b**) Using first order bidirectional Raman amplification (0.6 W Raman power launched on each end of the fibre) (from [29]); (**c**) Using a ultra-long Raman fibre laser cavity (URFL), using 0.7 W of Raman pump at 1365 nm assisted by a 1455 nm FBG on both ends of the fibre @2017 IEEE [32].

Figure 7 shows a comparison of the optical trace power along 125 km of fibre (a) without Raman amplification, (b) with first order bidirectional Raman and (c) over a URFL cavity (using second order Raman amplification) [32]. With the use of Raman amplification, the measurable range can be extended from a few tens of km to more than 100 km. While the use of second order Raman pump schemes provide a better trace optical power flatness, note that these typically also require higher pump powers and complexity.

#### Distributed Raman Amplification in CP-ΦOTDR

While several works claim to reach sensing ranges of >100 km [27,28,29,30,31,32] using distributed optical amplification, the impact on the sensor strain sensitivity/reliability is often not quantified. In addition, the use of excessively high Raman pump powers can render the system unpractical for field applications (e.g., using optical connectors).

**Figure 8:**
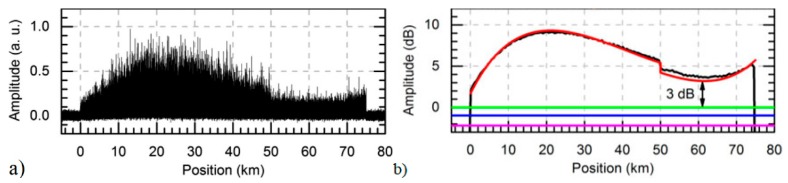
(From [27]) (**a**) Optical trace of CP-ΦOTDR using bidirectional first order Raman amplification over 75 km and (**b**) Corresponding optical SNR showing a minimum of 3 dB, allowing for a 1 nε sensitivity (there defined by strain standard deviation, see Equation (13)) along the fibre, @2017 IEEE [27].

In 2017, a configuration of CP-ΦOTDR using bidirectional first order Raman amplification over 75 km was proposed ([27], Figure 8). By ensuring a minimum of 3 dB optical SNR along the 75 km, the system allowed for a 1 nε strain sensitivity (there defined by the strain standard deviation (see Equation (13)), and limited by the digitizer quantization error) along the entire fibre, while maintaining the used Raman pump powers (230 mW co-propagating and 350 mW counter-propagating) well within the typical optical connectors specifications.

This early proof of concept attracted some criticism due to the high electric sampling used (40 GS/s sampling rate). However, it should be noted that this was a simple concept demonstration, and that similar results can be achieved using much lower sampling rates (see Section 5, where tens-hundreds pε/√Hz sensitivities (≈nε strain standard deviations; see Equation (13)) along 75 km are demonstrated using 1 GS/s of sampling and digital interpolation in the TDE, with real time processing).

As discussed later in Section 5, depending on the intended sensor performance and measurement parameters, CP-ΦOTDR can typically measure 30–50 km without distributed amplification. With the use of first-order Raman amplification (and depending on the used Raman pump powers), CP-ΦOTDR configurations exceeding 100 km are feasible while maintaining tens-hundreds pε/√Hz sensitivities (≈nε strain standard deviations; see Equation (13)). The use of higher order Raman amplification schemes should allow for higher measurable distances with similar performances (note that CP-ΦOTDR is particularly robust against RIN noise, since it measures temporal displacements, and is therefore is not directly affected by intensity fluctuations of the optical trace), however, such study is yet to be performed.

## 4. Strain Signal Properties

### 4.1. Laser Noise

#### 4.1.1. Laser Phase/Frequency Noise Affecting Strain Measurement

In CP-ΦOTDR, the presence of random phase noise $\varphi_{r}(t)$ in the laser source is translated into strain noise, since deviations in the nominal central frequency of emission produce an effect in the optical trace which is fundamentally indistinguishable from physical perturbation applied to the fibre [15]. In particular, each pulse (launched at $t_{i}$) experiences a different frequency drift ($\upsilon_{r}\left(t_{i}\right)=(1/2\pi )\cdot \partial \varphi_{r}(t_{i})/\partial t$ [11]) which affects the trace in an equivalent way to a perturbation-induced spectral shift Δυ. In other words, the shot-to-shot laser frequency drift $\upsilon_{r}\left(t_{i}\right)$ is equivalent to a certain constant strain ($\Delta \varepsilon_{r}\propto \upsilon_{r}\left(t_{i}\right)$) applied over the whole fibre and will be linearly added to the strain measurement as spatially correlated noise (Equation (6)).

It can therefore be derived [11] that the power spectral density (PSD) of the strain ($S_{\varepsilon }$) added to the measurement due to laser phase noise will be:

(9) $$S_{\varepsilon }=S_{\upsilon r}{\left(0.78\cdot \upsilon_{0}\right)}^{2}\propto \Delta f,$$

i.e., proportional to the laser random instantaneous frequency noise PSD ($S_{\upsilon r}$), which in turn is proportional to the laser static linewidth (Δf) [34].

**Figure 9:**
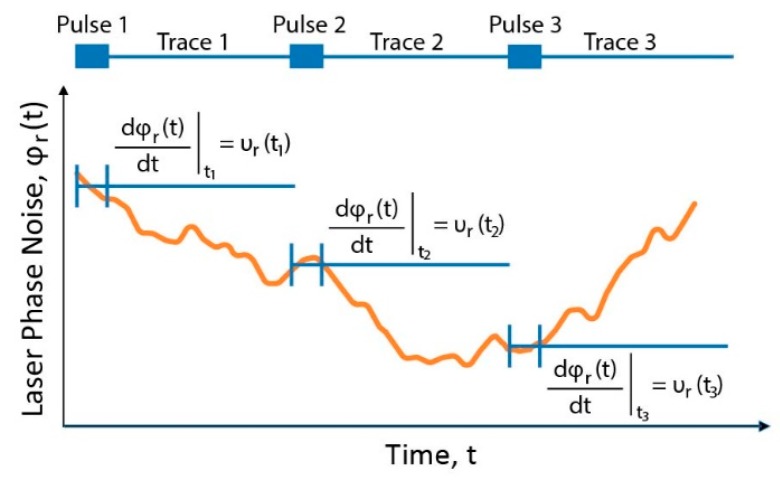
Effect of random laser phase noise $\varphi_{r}(t)$ over the trace length. In CP-ΦOTDR (blue line), the shot-to-shot frequency drift of the laser ($\upsilon_{r}\left(t_{i}\right)=(1/2\pi )\cdot \partial \varphi_{r}(t_{i})/\partial t$) induces a constant perturbation ($\Delta \varepsilon_{r}\propto \upsilon_{r}\left(t_{i}\right)$) along the whole fibre trace for each pulse/trace. In coherent-detection ΦOTDR schemes (orange line), the $\varphi_{r}(t)$ is randomly added to all points of the optical trace.

In coherent-detection ΦOTDR, the random laser phase noise $\varphi_{r}(t)$ will also be added as noise to the strain measurement. However, there is a key difference between these systems and CP-ΦOTDR: In CP-ΦOTDR $\varphi_{r}(t)$ will affect equally all measured points of the fibre ($S_{\varepsilon }$ is translated to an instantaneous frequency shift of the optical pulse which interrogates all the fibre). Meanwhile, in coherent-detection ΦOTDR, $\varphi_{r}(t)$ will affect all points of the fibre differently (in the detection process using the laser as a local oscillator, $\varphi_{r}(t)$ will be added continuously—and therefore randomly—along the optical trace), see example in Figure 9.

In this case, in CP-ΦOTDR the laser noise can be easily and effectively compensated without requiring external characterization of the laser noise (since it is already characterized in the fibre measured, as described below), which is not possible in traditional coherent-detection ΦOTDR.

#### 4.1.2. Laser Noise Compensation

The problem of cross-talk between laser noise $\varphi_{r}(t)$ and strain applied to the fibre can be solved by noting that a laser frequency drift $\upsilon_{r}\left(t_{i}\right)$ will affect all points of the fibre equally (Figure 9). Hence, the acquired set of data will show a perturbation affecting the entire fibre equally, but different from trace to trace. This perturbation is patent even in unperturbed fibre sections [11].

Based on this argument, a simple but effective method has been proposed to compensate the $\varphi_{r}(t)$ in CP-ΦOTDR. The laser frequency drifts $\upsilon_{r}\left(t_{i}\right)$ occurring from shot-to-shot are measured by computing the average temporal shift occurring in the optical trace along an unperturbed “reference” fibre section (Equation (5)):

(10) $$-\frac{\tau_{p}}{\delta \upsilon_{p}}\upsilon_{r}\left(t_{i}\right)=\langle \mathrm{TDE}\;\mathrm{along}\;\mathrm{reference}\;\mathrm{fibre}\rangle$$

This laser frequency drift $\upsilon_{r}\left(t_{i}\right)$ is then compensated for each instant for all points:

(11) $$\Delta t\left(x,i\right)=-\frac{\tau_{p}}{\delta \upsilon_{p}}\left(\Delta \upsilon \left(x,i\right)-\upsilon_{r}\left(t_{i}\right)\right).$$

The length of the reference fibre section simply needs to be large enough so that local perturbations/noise are averaged out to a noise level below the level of noise of each individual point [35]—A condition which is already met in typical operation. In practice, this fibre section length can be as small as 100 m [11].

**Figure 10:**
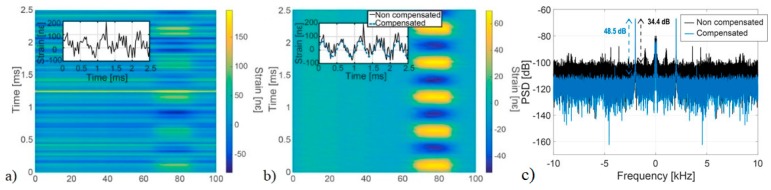
(From [11]) Strain measurements of a sinusoidal vibration applied to 20 m of fibre. The laser has a linewidth of 5 MHz and the induced phase noise was compensated using a 100 m unperturbed fibre. (**a**) Measured strain impaired by laser noise; (**b**) strain after laser noise compensation; (**c**) PSD of the measured strain signal, showing a 14 dB noise floor improvement after laser noise compensation, @2018 IEEE [11].

Figure 10 (from [11]) presents an experimental demonstration of this technique, where the noise of a 5 MHz linewidth laser is compensated using an unperturbed reference fibre section of 100 m. The experiment showed a 14 dB PSD noise floor improvement, thus illustrating the validity of the technique. However, the improvement was limited due to the existence of additional noises.

Recent measurements have demonstrated that this method can effectively provide a laser-noise-free measurement along the entire fibre, limited only by the additive noise in detection (see Section 5): tens pε/√Hz sensitivity measurements (with a PSD noise floor improvement of three orders of magnitude with respect to the noise floor set by laser noise) can be achieved, even when lasers presenting shot-to-shot frequency drifts of several MHz are used. In this case, the requirements for the used laser in CP-ΦOTDR are reduced to the use of a laser with a coherence length larger than the used optical pulse width $\tau_{p}$, without sacrificing the sensor’s performance.

### 4.2. Theoretical Strain Sensitivity Limit

#### 4.2.1. TDE Problem in Intensity CP-ΦOTDR

In CP-ΦOTDR, any given perturbation is translated to a local time-delay Δt of the retrieved photodetected optical trace. Therefore, computing the measurand at any given position within the fibre comprises a time delay estimation (TDE) problem [36], consisting of the determination of the delay between sections of the optical traces ($I_{0}$, $I_{i}$) photodetected at different instants, $t_{0}$, $t_{i}$ (Equation (5)). This approach is fundamentally different from traditional implementations of coherent-detection ΦOTDR, leading to different considerations (e.g., the common problem of fading points is absent in CP-ΦOTDR) and performance limits.

The TDE problem has been extensively researched due to its applicability in different fields, such as radar/sonar [37], and studies characterizing performance limits for different systems are readily available in the literature [38]. The Cramér-Rao Lower Bound (CRLB) for an unbiased time-delay estimator sets a limit for the mean-square error of TDE [12,38]. In principle, this may be obtained using a minimum variance unbiased estimator such as the generalized cross-correlation algorithm (i.e., finding the lag at which there is maximum correlation).

The CRLB for a CP-ΦOTDR system has been derived, taking into account the properties of the signals involved. Specifically, the CRLB derivation was based on the analytical developments in [38] for an active system, considering a triangular-shaped electric signal spectrum (note that the spectrum of the optical trace is rectangular, as described in Section 2, Equation (4), and the spectrum after photodetection is therefore triangular), with white noise across the signal band.

In this case, the limit for the strain variance ($\sigma_{CRLB,\varepsilon }^{2}$) in CP-ΦOTDR is given by:

(12) $$\sigma_{CRLB,\varepsilon }^{2}=\frac{3}{{\left(\left(0.78\right)2\pi \right)}^{2}}\frac{1}{SNR_{electrical}}\frac{1}{\upsilon_{0}^{2}\cdot \delta \upsilon_{p}\cdot \tau_{corr}^{3}},$$

for a particular trace electrical SNR ($SNR_{electrical}$), cross-correlation time window $\tau_{corr}$ (assumed to be equal to the pulse width, $\tau_{p}$, in order to optimize measurand spatial resolution), chirp-induced bandwidth $\delta \upsilon_{p}$ and where $\upsilon_{0}$ is the center frequency of the laser probe pulse.

Equation (12) essentially determines the minimum variance for a strain measurement, which translates to a spectrally white noise floor. This limit may be reached when operating under ideal conditions, i.e., with enough trace SNR and time-bandwidth product to mitigate the occurrence of large errors [19] (typically $SNR_{electrical}$ > 3 dB, see experimental section), presenting purely additive noise in detection, and without impairment from other noise sources (such as laser phase noise, quantization noise or instrument jitter). These results are valid for small applied strains (i.e., those inducing a frequency shift of up to 3–5% of the pulse spectral content, see Section 4.3 for discussion on large strains), so that trace distortion and decorrelation may be neglected. For a given acoustic sampling ($f_{s,ac}$—limited by the length of the fibre, see Equation (7)), the strain amplitude spectral density (ASD) noise floor $ASD_{noise\;floor}$, is then given by:

(13) $$ASD_{noise\;floor}=\sigma_{CRLB,\varepsilon }/\sqrt{f_{s,ac}/2}\;\varepsilon /\sqrt{Hz}$$

where $\sigma_{CRLB,\varepsilon }$ is the CRLB limit for strain standard deviation. Note that in the literature, the sensor sensitivity is sometimes described by the strain standard deviation (units of ε), rather than by the amplitude spectral noise (units of $\varepsilon /\sqrt{\mathrm{Hz}}$).

**Figure 11:**
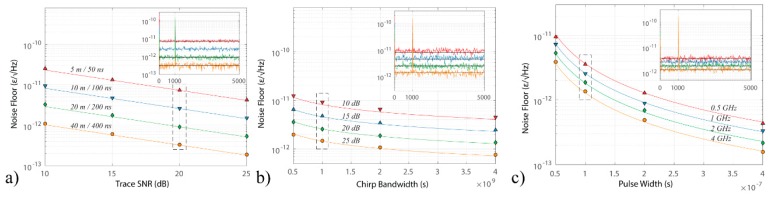
(From [12]) ASD strain noise floors of simulated data and for varying parameters of Equation (12). (**a**) Noise floor as a function of trace $SNR_{electrical}$ and $\tau_{p}$. (**b**) Noise floor as a function of $\tau_{p}$ and $\delta \upsilon_{p}$. (**c**) Noise floor as a function of $\delta \upsilon_{p}$ and trace $SNR_{electrical}$, @ 2018 IEEE [12].

The relation described by Equation (12) has been numerically and experimentally verified. In the numerical test, an optical trace obtained from a 400 m section of fibre with purely additive noise in detection has been simulated. The strain noise floor was then determined for varying parameters of Equation (12), as presented in Figure 11 (from [12]). In all cases, an excellent match with the theoretically expected results has been obtained.

**Figure 12:**
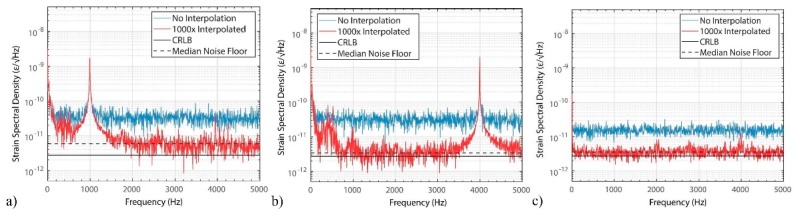
(From [12]) Strain ASD from a 0.2 s acquisition with (**a**) a 1 kHz sinusoidal perturbation (Estimated trace $SNR_{electrical}$ = 19.47 dB. CRLB calculated at 2.715 × 10^−12^ ε/√Hz, median noise floor measured at 5.178 × 10^−12^ ε/√Hz); (**b**) a 4 kHz sinusoidal perturbation (Estimated trace $SNR_{electrical}$ = 19.38 dB. CRLB calculated at 2.744 × 10^−12^ ε/√Hz, median noise floor measured at 3.421 × 10^−12^ ε/√Hz); (**c**) in a thermally stable section of the fiber (Estimated trace $SNR_{electrical}$ = 19.62 dB. CRLB calculated at 2.668 × 10^−12^ ε/√Hz, median noise floor measured at 3.590 × 10^−12^ ε/√Hz), @ 2018 IEEE [12].

Experimentally, reaching the theoretical limit of Equation (12) turns out to be a feasible task. In [12], a record sensitivity median noise floor of 3.421 × 10^−12^ ε/√Hz has been attained, only 20% away from the CRLB (Figure 12). The small differences between the measured and expected noise floor may stem from error in the local trace $SNR_{electrical}$ estimation, originating from the stochastic nature of the acquired backscatter power-trace.

#### 4.2.2. Minimum Required Optical Trace SNR

Adding to the reliability (low sensitivity variance across sensing channels) of the system, the TDE-based measurement of CP-ΦOTDR has also proven to be a robust technique against optical trace noise. While there is currently no statistical model to predict the occurrence of large errors under a given trace SNR, this has been experimentally measured. Under typical operation, reliable strain measurement (with high sensitivities and a negligible probability of large errors) towards the end of the fibre is possible when the average trace $SNR_{electrical}$ is as low as 3 dB (see experimental Section 5: the occurrence of large strain errors does not have a relevant impact until trace $SNR_{electrical}$ ~≤ 3 dB in all configurations). Since the technique relies on optical intensity, averaging can also be readily used to extend the measurable range into fibre regions where the single-shot trace SNR is <0 dB. Hence, this averaging process can be either temporal (i.e., by averaging successive traces), or spatial (i.e., by increasing the correlation window). Still, further research should be done on the effects of averaging successive traces, e.g., how averaging may affect the strain measurement, as well as the averaged trace bandwidth in the presence of jitter and/or laser phase noise.

### 4.3. Maximum Measurable Strain: Shot-to-Shot Limit

Similarly to other Rayleigh-based distributed sensors, CP-ΦOTDR measures changes in the strain (and/or temperature,) applied to the fibre relative to a previously acquired reference state. However, there is a limit to the relative value of Δε applied to the fibre that can be appropriately measured. For the measurement of arbitrarily large and unknown perturbations, the possibility of performing a previous fibre scan as an initial reference for all subsequent traces may not always be feasible, as this would require an impractical laser frequency sweep and/or unachievably high pulse spectral content. Note that the equivalent $\delta \upsilon_{p}$ for an applied strain of 1000 µε is 150 GHz. The issue of temporal stability of said references would also need to be addressed.

Therefore, CP-ΦOTDR, similarly to other dynamic ΦOTDR schemes, relies on the use of differential (temporal) strain measurement, i.e., the strain is computed relative to the previous acquisition, and the strain signal is obtained by integrating the strain variations over time. Note that this differentiation refers only to the time domain. In the spatial domain, each spatial point is computed independently (without differentiation or crosstalk) from its neighboring points.

In this case, the measurement is limited by the maximum measurable strain variation shot-to-shot, $\Delta \varepsilon_{\max}$. Therefore, the measurable dynamic strain is limited not in absolute amplitude, but rather as a maximum measurable strain variation rate, $\Delta \varepsilon_{\mathrm{rate},\max}$, defined by:

(14) $$\Delta \varepsilon_{rate,\max}=f_{s,ac}\cdot \Delta \varepsilon_{\max}.$$

Note that the maximum measurable $\Delta \varepsilon_{\max}$ is limited due to the occurrence of large errors (also known as outliers) in the TDE [12,19,20,39] for large strain variations. The appearance of large errors is a statistical phenomenon and cannot be fully eliminated (although it can be removed in post-processing). While no in-depth study has been performed to characterize this effect in the specific case of CP-ΦOTDR, it has been empirically found that its occurrence can be greatly reduced if $\Delta \varepsilon_{\max}$ is maintained below the equivalent to 3–5% [39] of the pulse spectral content, i.e.,:

(15) $$\Delta \varepsilon_{\max}=\frac{1}{0.78}\left(\alpha \frac{d\upsilon }{\upsilon_{0}}\right),$$

where typically α = 0.03−0.05. Under optimal optical SNR conditions, operation with α of up to 0.1 (i.e., $\Delta \varepsilon_{\max}$ equivalent to 10% of pulse spectral content $\delta \upsilon_{p}$) can be performed.

**Figure 13:**
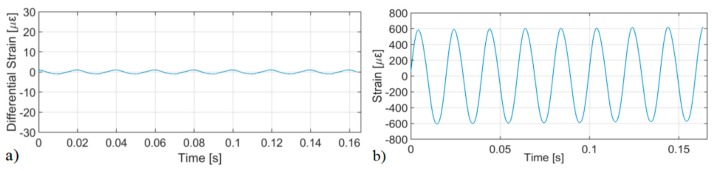
(From [39]) Measurement of large strain (50 Hz, 1190 µε (peak-to-peak) vibration) using CP-ΦOTDR, $\tau_{p}$ = 35 ns (3.5 m spatial resolution), $\delta \upsilon_{p}$ = 5 GHz and $f_{s,ac}$ = 200 kHz, after 5-point median filter of differential strains: (**a**) shot-to-shot differential strain (**b**) measured strain, @2019 IEEE [39].

Full scalability of this measuring principle for large strain has been demonstrated by measuring 50 Hz perturbations with an amplitude >1000 µε (Figure 13). This experiment relied on an acoustically oversampled acquisition (high $f_{s,ac}$) of the perturbation, in order to limit the maximum strain between two consecutive measurements (see Equation (14)). If the probability of large errors is sufficiently low to ensure sparsity, it was also shown that they may be easily removed via a simple median filter applied to the differentiated strain signal (further exploiting the acoustic over-sampling), resulting in a measured signal which matches the applied perturbation without artifacts and high harmonic rejection (>30 dB) [39]. In this case, $\Delta \varepsilon_{\max}$ was ≈1 µε (equivalent to ≈3% of $\delta \upsilon_{p}$).

### 4.4. Reliability and Sensitivity Variability (Fading Free Measurement)

The statistical nature of the backscattered power traces in ΦOTDR ensures the periodical existence of points where the optical intensity falls below the noise of the system—so-called fading points. In these fading points, a reliable optical phase (and therefore strain) measurement cannot be performed. More generically, in traditional coherent-detection ΦOTDR the error in the strain measurement will vary greatly from point to point, even for neighbouring spatial points, depending on the (random) amplitude of the trace in each point:

(16) $$SNR_{dynamic}(i,i\prime )=\frac{2\sigma_{\phi }^{2}}{\sigma_{n}^{2}\left[1/A^{2}\left(i\right)+1/A^{2}\left(i\prime \right)\right]},$$

where $\sigma_{\phi }^{2}$ is the variance of the detected phase signal, $\sigma_{n}^{2}$ is the noise variance and $A^{2}\left(i\right)$, $A^{2}\left(i\prime \right)$ are exponential-distributed random processes associated to the detected backscattered profile. While works describing and addressing these issues have been presented [40,41,42,43], fading points remain an ever present issue to be accounted for in traditional coherent-detection (relying on phase measurement) schemes.

In the case of CP-ΦOTDR, however, when computing time delay estimations (TDE) along the optical fibre trace, the lateral displacement of a point of optical intensity equal to 0 can be computed in the same manner as the lateral displacement of a point with high optical intensity (see Figure 1). Therefore, as long as the visibility of the trace is well conditioned, the technique essentially provides a fading free measurement along all points of the fibre, thus surpassing one of the main problems of DAS (fading points leading to high sensitivity variability from spatial point to point).

**Figure 14:**
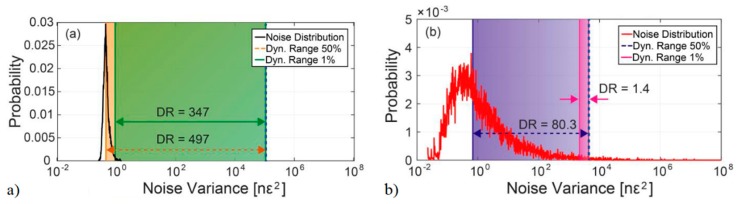
(From [44]): Noise distribution and dynamic range of the sensor: (**a**) CP-ΦOTDR; (**b**) coherent-detection ΦOTDR. Vertical dashed lines at the right point out the maximum shot-to-shot measurable perturbation. Green and pink shaded rectangles mark the limits of dynamic range assuming 1% of noisy measurements (SNR ≤ 1); yellow and purple rectangles mark the limits of dynamic range for 50% of noisy measurements, @2018 IEEE [44].

Figure 14 presents a comparison of the strain noise and dynamic range (i.e., ratio between maximum measurable strain from shot-to-shot and strain noise floor) for (a) CP-ΦOTDR; (b) coherent-detection ΦOTDR. It should be noted that in CP-ΦOTDR the strain noise floor is a well defined parameter with low variability. In coherent-detection ΦOTDR, however, that definition depends on the degree of trustworthiness required for the measurement, varying by orders of magnitude if it is defined to provide a trustworthy measurement over 50% of the fibre points or 99% of the fibre points. Regarding the experiment, it was performed with probe pulses of similar peak power and width and under typical experimental conditions (from [44]). The differences in the experiment simply reflected the intrinsic differences of the two techniques: the use of a chirped pulse (higher spectral content) and correspondingly higher detection bandwidth in the case of CP-ΦOTDR.

The presented results fully verify the reliability of CP-ΦOTDR. With similar nominal sensitivities for both cases (defined as the mean strain SNR [45]), the variability of the strain noise of CP-ΦOTDR is low, while in the case of the coherent-detection-based sensor, even with a good average strain SNR, the sensor will always present points with low strain SNR (<1), which impair the reliability of the system.

In practice, this means that CP-ΦOTDR allows for trustworthy strain measurements with broad dynamic ranges in all positions of the fibre. Typically dynamic range is >300: sensitivity noise floor at high frequencies is typically below 1 nε (defined by strain standard deviation, see Equation (13)) under good trace SNR conditions (see Section 5) and $\Delta \varepsilon_{\max}$ is typically 100′s of nε (note that the Δε inducing a Δυ of 5% of 1 GHz is ~330 nε).

### 4.5. Long Term Stability and Temperature Measurements

The discussion on long-term stability (days/weeks) in ΦOTDR (in general), is an issue rarely addressed in the literature. An in-depth discussion on the use of fibre pre-calibrations in ΦOTDR (recording the fibre “finger-print” for all possible states) is out of the scope of this paper. However, it should be noted that with an operation which integrates the variation of strain/temperature accumulated over time, ΦOTDR sensors provide high measurand sensitivities but will inevitably accumulate measurement errors over time. Therefore, an absolute stability of temperature/strain measurements over months/years (as achieved by Brillouin based sensors [46]) is not expected.

**Figure 15:**
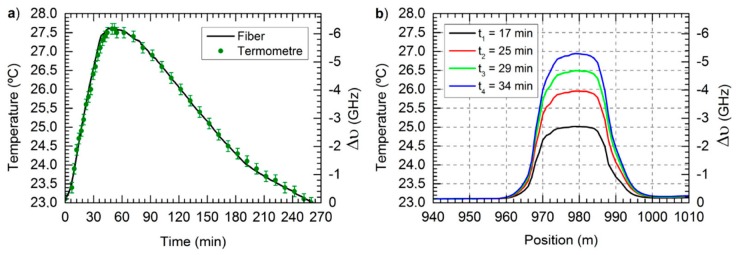
(From [10]) Measured temperature variations when temperature is raised from 23 °C to 27.5 °C and back to 23 °C in 20 m of fiber around meter 979 of the FUT, over 270 min. (**a**) Temperature evolution of meter 979 along time (**b**) Temperature profile along 70 m of fiber at different times. @2016 The Optical Society [10].

In CP-ΦOTDR, the initially presented results [10] readily demonstrated temperature errors accumulated along several hours below the 0.1 K thermometer error (Figure 15), for temperature variations of several degrees.

**Figure 16:**
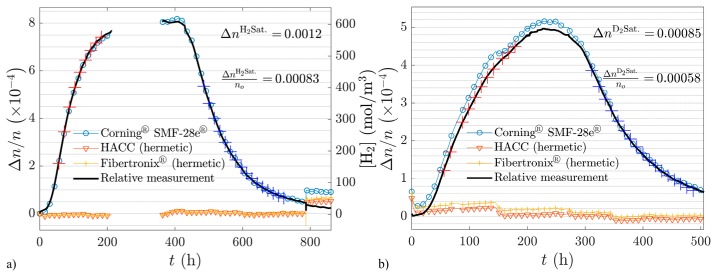
(From [47]) (**a**) Relative refractive index evolution due to the hydrogen loading (150 bars) as registered at the different fiber samples. The difference between the permeable and one of the hermetic fibers is shown (relative measurement); (**b**) equivalent deuterium experiment (100 bars). The red and blue crosses represent exponential fittings of the relative measurement curves. @2019 SPIE [47].

Recently, the feasibility of CP-ΦOTDR for long measurement has been demonstrated in an experiment that lasted for several weeks, aimed at detecting/quantifying the presence of hydrogen/deuterium in optical fibres, by monitoring the correspondent refractive index variation of the fibre [47]. The main results are illustrated in Figure 16 (see caption for details). A quantification of the measurement error was not possible due to the lack of a reference measurement (with another technique) to compare the results to but the results qualitatively matched the expected refractive index variation, demonstrating the long-term stability of CP-ΦOTDR. To the best of our knowledge, this is the longest experiment continuously measuring a perturbation with ΦOTDR sensor.

Regarding the measurement of slow strain (e.g., the measurement of Earth tides, of relevance for seismology, translates into strain variations over 12–24 h), while laboratory measurements can be performed, it should be noted that an important issue for practical applications concerns the temperature cross-sensitivity. Note that a temperature change of 10 mK is in principle indistinguishable from a ≈90 nε variation (Equation (6)), and maintaining/measuring such temperature variations along several km of fibre is an unpractical scenario in most applications.

## 5. Experimental Results

Since its development, extensive research on CP-ΦOTDR has been presented, including several studies discussing specific performance parameters (e.g., sensing range, maximum achievable sensitivity or dynamic range), thus characterizing and demonstrating the potential of the technique. However, the direct comparison of said works may not be straightforward, since different measurement settings and/or optical components were used. For example, the reader may wonder whether the maximum sensitivity may be achieved for the maximum sensing range, or whether the CRLB is achievable for lower digital samplings or with different optical filters.

In this section, the work of [10,11,12,27,44] is extended, and the performance of CP-ΦOTDR (mainly sensing range and noise) is characterized for different measurement settings using a single optical setup, thus providing a general overview of the technique. The complexity/cost of the sensor is also greatly reduced by employing widely available, low cost components, instead of high performance laboratory equipment (which were previously used for concept demonstrations, but are not required to achieve a high performance sensor).

### Experimental Setup

**Figure 17:**
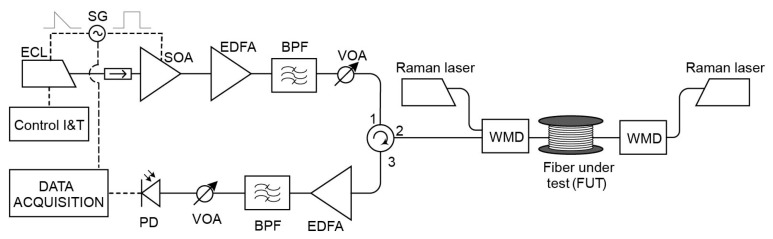
Experimental setup used for the measurements of this section, based on that of the initial CP-ΦOTDR demonstration [10]. ECL: External cavity laser; SG: Signal generator: I&T: Intensity and temperature; SOA: Semiconductor optical amplifier; EDFA: Erbium doped fibre amplifier; BPF: Band-pass filter; VOA: Variable optical attenuator; WDM: Wavelength division multiplexer; PD: Photodetector. Solid line represents optical path, dashed line represents electrical path.

The experimental setup (Figure 17) is based on that of the initial CP-ΦOTDR demonstration [10]. An external cavity laser (ECL), with 1 MHz linewidth, operating in continuous emission is used as light source. The laser central wavelength is 1550 nm ($\upsilon_{0}$ = 193.40 GHz), controlled by an external current and temperature controller. A secondary current control is used to introduce repetitive current ramps, thus introducing a periodic linear chirp in the outputted laser light. The laser output light is then pulsed in the time-domain using an SOA with nominal 50 dB extinction ratio and rise/fall times in the order 2.5 ns, fed with rectangular-like electrical pulses synchronized (same signal generator) with the laser chirp ramps. The pulse repetition rate ($f_{s,ac}$) is 1 kHz, allowing measurements of up to ~100 km of fibre (Equation (7)). The output optical pulses present a 100 ns FWHM ($\tau_{p}$—setting a spatial resolution of 10 m) and 400 MHz spectral content ($\delta \upsilon_{p}$). An amplification stage composed of an EDFA, a 100 GHz standard dense wavelength division multiplexer (DWDM) (used as BPF), and a VOA is used to control the pulse peak powers before launching then into the FUT via an optical circulator. For the experiment, three FUTs are used with 50 km (single 50 km fibre roll), 75 km (50 km + 25 km fibre rolls), and 100 km (50 km + 50 km fibre rolls) of SMF. A calibrated piezoelectric (with a length of 60 m) is used to apply sinusoidal strain signals of known amplitude. The setup allowed the possibility of using distributed Raman amplification (with no amplification, co-propagating amplification or bi-directional amplification) with the use of the Raman pump lasers (emitting at 1455 nm, allowing pump powers of up to 400 mW each), coupled to the FUT via 1450/1550 WDMs [27]. Another amplification stage (composed of an EDFA, a 100 GHz standard DWDM, and a VOA) was used to amplify the FUT Rayleigh backscattered light, before reaching the photodetector (520 MHz bandwidth p-i-n). The optical trace was recorded with a 1 GS/s digitizer and processed by a commercial GPU. All data was processed in real-time, aiming at demonstrating the practical feasibility of the sensor, and streamed to an external disk via a common USB 3.0 port.

With respect to previous experiments the cost/complexity of the system was greatly reduced while maintaining the high performance of the sensor. The sensor did not require [12] the use of high coherence lasers (replaced by low coherence laser using laser noise compensation [11]) and/or external modulator controlled by an arbitrary waveform generator (AWG): the laser was directly modulated in current. The BPFs are low-cost commercially available and the use of high sampling rate digitizer was avoided (1 GS/s with digital interpolation [12], replacing the 40 GS/s used for the initial concept demonstration [10]).

With the sensor presented in Figure 17, the optical traces and strain variation signals were recorded for all positions of the fibre during a statistically significant time (2–5 min), allowing for distributed characterization of trace SNR and strain noise/sensitivity characterization along the entire fibre link.

**Figure 18:**
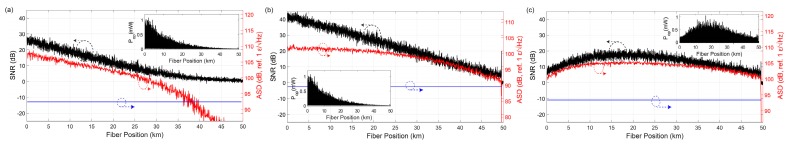
Fibre trace $SNR_{electrical}$ (black), inverted strain $ASD_{noise\;floor}$ (red), and equivalent laser noise level (blue) along 50 km of fibre, for three different measurement settings (with 100 ns FWHM pulses ($\tau_{p}$) and 1 kHz pulse repetition rate ($f_{s,ac}$)): (**a**) Pulse peak power = 200 mW without Raman amplification, without averaging; (**b**) Pulse peak power = 200 mW without Raman amplification, with 40 averages of the optical trace; (**c**) Pulse peak power = 40 mW without averaging, co-propagating Raman pump = 300 mW. Insets show the photodetected traces.

Figure 18 shows the results for a 50 km fibre measurement with three different measurement parameters (see figure caption for details). The trace electrical SNR ($SNR_{electrical}$—black line) is computed by using the variance of the photodetected signal before the beginning of the trace as the noise level (N) and the photodetected trace power along 10 m windows (i.e., equal to the used correlation window $\tau_{corr}$, thus matching the sensor spatial resolution) as signal (S) [45].

The figure also shows the strain ASD noise floor ($ASD_{noise\;floor}$—sensor’s sensitivity noise floor) in each case (red line, inverted, for visual comparison with the $SNR_{electrical}$). With the used configuration, the theoretical CRLB limit for strain $ASD_{noise\;floor}$ (see Equations (12) and (13), see [12]) is:

(17) $$CRLB\;limit\;for\;ASD_{noise\;floor}=\sqrt{\frac{3}{{\left(\left(0.78\right)2\pi \right)}^{2}}\frac{1}{SNR_{electrical}}\frac{1}{\upsilon_{0}^{2}\cdot \delta \upsilon_{p}\cdot \tau_{corr}^{3}}\cdot \frac{2}{f_{s,ac}}}\;\varepsilon /\sqrt{Hz}\approx 2.89\cdot 10^{-9}\sqrt{\frac{1}{SNR_{electrical}}\cdot \frac{2}{f_{s,ac}}}\varepsilon /\sqrt{Hz}$$

The offset and scale between the axis of the $SNR_{electrical}$ and strain $ASD_{noise\;floor}$ are adjusted so that the theoretical CRLB limit of strain $ASD_{noise\;floor}$ would be the overlap of the red and black curves (according to Equation (17)). For an easier visualization, the results are presented in logarithmic scale and referenced to 1 pε/√Hz, using: $ASD_{noise\;floor}[\mathrm{dB}]=10\cdot \log_{10}(ASD_{noise\;floor}/[1\;\varepsilon /\sqrt{\mathrm{Hz}}])$ (e.g., $1\;n\varepsilon /\sqrt{\mathrm{Hz}}=\;-90\;\mathrm{dB},\;\mathrm{ref}.\;\mathrm{to}\;1\;\varepsilon /\sqrt{\mathrm{Hz}}$).

A few comments can be made to the presented results. The similarity of the red and black curves (in terms of absolute value, as well as variation along the fibre), demonstrates that the presented CP-ΦOTDR is well conditioned, operating with performance levels close to the theoretical CRLB limit level. Regarding Figure 18a, a sensitivity of 15 pε/√Hz is achieved at the fibre beginning and the sensing range is limited to ≈35 km (note that the noise increases rapidly after this point) due to the occurrence of large errors when the trace SNR falls below a certain level. In any case, the robustness of the TDE via use of correlations in CP-ΦOTDR is well demonstrated, allowing operating with 100 pε/√Hz, even when the trace $SNR_{electrical}$ is as low as 3 dB (for this configuration; note that operation below 0 dB SNR is possible for larger chirps $\delta \upsilon_{p}$ and/or correlation windows $\tau_{corr}$). Regarding Figure 18b, it is observed that the sensing range can be extended with the use of trace (temporal) averaging, as the entire 50 km of FUT is measured. The averaging is performed by acquiring and averaging the photodetected signal of N (=40) consecutive traces before performing the TDE (i.e., strain calculation). Note that in this case the frequency response of the system is changed, as averaging will act as a low pass filter in the acoustic response, i.e., there is a trade-off between acoustic bandwidth and sensing range. The improvement of the strain $ASD_{noise\;floor}$ for increasing trace SNRs saturates when $SNR_{electrical}\approx 25\;\mathrm{dB}$, (red line deflects from theoretical CRLB close to the fibre beginning), indicating the presence of additional residual noises in the system. With the use of distributed amplification (Figure 18c), the performance along the 50 km is homogenized, with the strain $ASD_{noise\;floor}$ being keep above 100 $p\varepsilon /\sqrt{Hz}$ in all fibre. With the use of averaging (without Raman)—Figure 18b—or Raman (without averaging)—Figure 18c—the sensing range is extended beyond 50 km.

**Figure 19:**
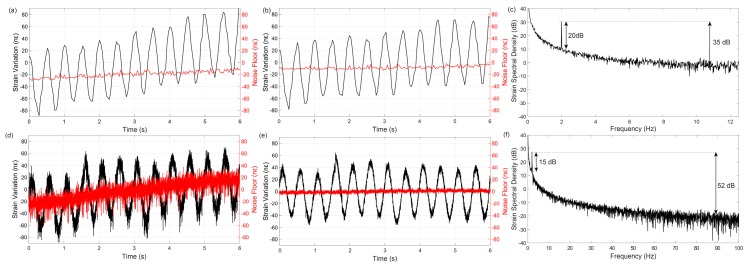
Strain signal at the piezoelectric fibre section (black), placed at the end of the fibre (50 km—point of lowest SNR), applying a 2 Hz sinusoidal strain signal with 80 nε peak-to-peak. In red, a neighboring channel (placed at 49 km), showing the measurement noise floor for comparison. Strain measured with configurations of Figure 18b (**a**–**c**) and Figure 18c (**d**–**f**) is presented; The time-domain of the strain signal is shown before (**a**,**d**) and after (**b**,**e**) laser noise compensation; (**c**,**f**) Strain PSD for the applied perturbation.

**Figure 20:**
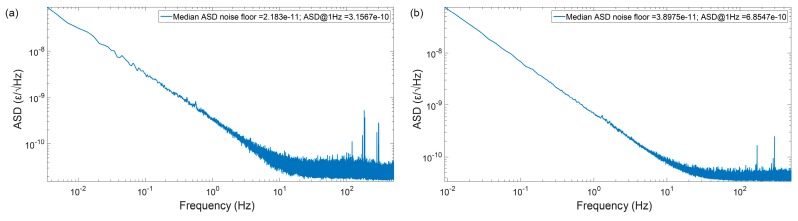
Average strain ASD for all the fibre points, during 5 min for (**a**) the first 10 km using configuration of Figure 18a (200 mW pulse power, without the use of Raman or averaging), (**b**) for the entire 50 km using configuration of Figure 18c (40 mW pulse power, without the use of averaging, 300 mW co-propagant Raman pump).

The laser noise level, (i.e., the strain noise level of the measured signal before compensating the laser noise) is presented in blue, demonstrating the effectiveness of the technique [11]: an improvement of up to 15 dB in strain $ASD_{noise\;floor}$ (i.e., 30 dB in strain PSD) is achieved by compensating the laser noise. This improvement (as well as a demonstration of the performance of the sensor after 50 km of fibre) is also shown in Figure 19: a 2 Hz sinusoidal strain signal with 80 nε peak-to-peak applied by the piezoelectric is displayed before and after the laser noise compensation. Small but noticeable temperature drifts are also observed. See Figure caption for details. By computing the PSD of the strain signal, an SNR of 20 dB for configuration of Figure 18b (presented in Figure 19c) and an SNR of 15 dB for configuration of Figure 18c (presented in Figure 19f) is measured for this perturbation. However, it should be noted that the acoustic SNR at this frequency (2 Hz) is impaired by the existence of 1/f noise in the measurement (see Figure 20 for further details on this noise). Perturbations of similar amplitude at higher frequencies would yield an SNR of up to 35 dB and 52 dB, respectively, as depicted.

Figure 20 shows the strain ASD distribution, for the first 10 km of fibre (of configuration of Figure 18a) and for the entire 50 km of fibre (of configuration of Figure 18c). Note that the results show the average statistics for all the fibre points (in the considered section), during 5 min. As expected, the best performance can be achieved at the beginning of the fibre, where the trace SNR is higher (Figure 20a), but with the use of distributed amplification the performance is homogenized along the fibre, achieving a high performance along the entire 50 km (Figure 20b). A strain ASD noise floor of 22 pε/√Hz (Figure 20a) VS 39 pε/√Hz (Figure 20b) is demonstrated at high frequencies. At lower frequencies (of relevance for e.g., seismic applications), a 315 pε/√Hz (Figure 20a) VS 685 pε/√Hz (Figure 20b) is demonstrated at 1 Hz.

Note that the main difference when comparing to the results of Figure 20a to [12] (over 10 km) is the use of a lower trigger rate ($f_{s,ac}$ = 1 kHz here VS 10 kHz in [12]), but the performances are comparable.

While an extensive discussion on the existing 1/f noise at low frequencies is out of the scope of this paper, it should be noted that the measurement was not performed in a thermally stabilized environment (i.e., it is unclear if this measurement is partially impaired by environmental noises at said frequencies, and not only the noise of the measurement technique).

**Figure 21:**
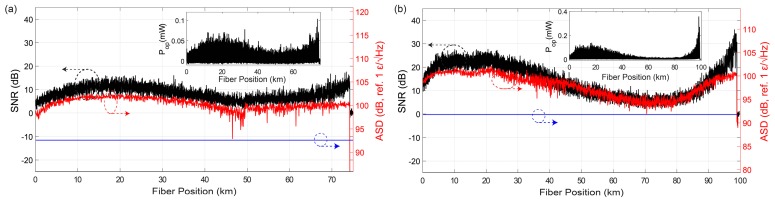
Fibre trace $SNR_{electrical}$ (black), inverted strain $ASD_{noise\;floor}$ (red), and equivalent laser noise level (blue), with 100 ns FWHM pulses ($\tau_{p}$) and 1 kHz pulse repetition rate ($f_{s,ac}$) along: (**a**) 75 km, pulse peak power = 30 mW, without averaging, bi-directional Raman amplification (co-propagating Raman pump = 270 mW, counter-propagating Raman pump = 245 mW); (**b**) 100 km, pulse peak power = 30 mW, with 20 averages of the optical trace, bi-directional Raman amplification (co-propagating Raman pump = 300 mW, counter-propagating Raman pump = 380 mW). Insets show the photodetected traces.

The possibility for long range measurements is characterized in Figure 21, where the trace $SNR_{electrical}$ VS strain $ASD_{noise\;floor}$ is computed along the FUT for 75 km and 100 km, using bi-directional Raman amplification (details in figure caption).

The sensor strain $ASD_{noise\;floor}$ is maintained above 150 pε/√Hz and 400 pε/√Hz, respectively (corresponding to a strain standard deviations of ≈3.3 nε and ≈2 nε, see Equations (13) and (17)) in the worst SNR point of the fibre in both cases, which demonstrated the high sensitivity of the sensor even for long ranges.

## 6. Comparison of Performance with Respect to Alternative Distributed Sensors

Typically, CP-ΦOTDR allows for reliable (fading free), dynamic (single-shot, with $f_{s,ac}$ at kHz-MHz rate), high sensitivity (down to pε/√Hz, with sub-nε strain standard deviations; see Equation (13)), quantitative (strain/temperature/refractive index) variations, with metric spatial resolutions, along optical fibres with tens of km to >100 km.

When compared to Brillouin based sensors (also capable of metric resolutions over 100 km and beyond [48]), BOTDA typically allows for microstrain resolutions with measurement times of a few minutes [46]. Although dynamic (kHz) versions of BOTDA have been proposed [49], the two techniques operate in different ranges. The main advantage of BOTDA is that it allows for an absolute stability of the measurement of temperature/strain applied to the fibre, which is expected to remain reliable after months/years. In the case of CP-ΦOTDR (and ΦOTDR in general), faster and more sensitive measurements can be achieved, but for variations of temperature/strain applied to the fibre, which will inevitable accumulate an error for long integration times.

Operating in the same applicability range, CP-ΦOTDR can be in general compared with other ΦOTDR based sensors. When using direct detection, traditional ΦOTDR allows for similar sensing range/acoustic sampling rate performances, but with a critical difference: the technique provides a nonlinear measurement, and therefore temperature/strain perturbations cannot be quantified, which greatly limits the performance of the sensor [17,29,32].

For linear/quantified measurements in ΦOTDR, coherent-detection [13,15,28,30,31,40,41,42,45,50,51,52,53] or a frequency sweep ΦOTDR [15,54,55] are required. In the case of frequency sweep, the achieved sensitivities are closer to the measurable by CP-ΦOTDR (equivalent to a few MHz frequency detuning: 10 mk [15]/refractive index variations of 10^−7^ [55]) for meter range spatial resolutions. Similarly to Brillouin based techniques, the requirement of a frequency sweep typically limits the measurement time to seconds-minutes, but presenting advantages for static measurements: the measurement of birefringence equivalent to a frequency detuning of 75 GHz was demonstrated in [55].

As for the case of ΦOTDR using coherent-detection, the technique is directly comparable to CP-ΦOTDR, with linear measurements achievable in single-shot operation. Regarding complexity, in ΦOTDR using coherent-detection, the laser requirements are increased due to the use of a local oscillator (while in CP-ΦOTDR high sensitivity measurements are demonstrated using 1 MHz linewidth lasers, and the chirp can be directly applied to the laser via current modulation, without requiring external modulator or AWG). As for the detection requirements, in CP-ΦOTDR, a single photodetector/digitizer is required, but with higher detection bandwidth, for the same spatial resolution (typically up to 1–2 GHz VS few hundred MHz used in coherent-detection ΦOTDR). In ΦOTDR, for the case of I/Q detection [13], a 90 °C hybrid and two photodetectors/digitizer channels (or even four, to solve the polarization fading issues using polarization diversity receiver [40]), are required.

But the biggest differences in operation between CP-ΦOTDR and coherent-detection based ΦOTDR are encountered in the reliability/trace SNR requirements of the two systems. The TDE-based measurement of CP-ΦOTDR allows for high reliability (low sensitivity variability across sensing channels) and robustness of the technique against optical noise (as demonstrated in this paper: high sensitivity strain measurements are obtained even for trace (envelop) SNRs of only a few dB). In coherent-detection based ΦOTDR however, the strain sensitivity can vary by orders of magnitude for neighboring channels, due to the existence of fading points (intrinsically associated with the random nature of ΦOTDR traces). While methods to mitigate this effect have been proposed (e.g., using inner-pulse frequency-division [40], which also demands higher pulse/detection spectral contents), the issue remains an added complexity to be solved. The use of I/Q detection also required higher trace (envelop) SNRs, since the phase unwrapping method presents impairments when trace SNR is low.

Regarding spatial resolution, CP-ΦOTDR presents a high trade-off dependency in this parameter (Equation (12)). On the one hand, operation in the sub-meter spatial resolution regime, while feasible (either with shorter pulses, or with the use of sub-band processing to increase the spatial resolution [18] beyond $\tau_{p}$), is expected to increase in complexity/cost for shorter resolutions, due to the requirement of using higher spectral contents $\delta \upsilon_{p}$. In this regime, schemes using matched filtering have been demonstrated to allow for high performance with sub-meter spatial resolutions [40,41,42,50,51,53,56,57,58], using typical spectral contents of up to ~1 GHz. Nevertheless, these solutions also add to the cost and complexity of the system, as they often require coherent detection with multiple high-bandwidth photodetectors and digitizers, as well as high quality pulse frequency modulation (typically using narrow linewidth lasers with external modulation and an AWG). Note that matched filtering is here mentioned in general as a set of techniques which can break the pulse width – spatial resolution ratio (e.g., optical pulse compression (OPC) based schemes), although the different techniques proposed in the literature vary in implementation/operation. Recently, an experiment with suppressed fading (with a minimum intensity trace SNR of 30 dB along the fibre) demonstrated 0.8 m spatial resolution with 245.6 pε/√Hz over 10 km [40].

On the other hand, with meter spatial resolutions over tens km to >100 km, CP-ΦOTDR can achieve high performances even with low complexity/cost setups (as demonstrated in this paper). With a spatial resolution of 10 m, CP-ΦOTDR has demonstrated (to the best of our knowledge) the highest sensitivity in distributed sensing [12]: 3.4 pε/√Hz over 10 km, almost reaching the theoretical CRLB limit. And even better performances are expected at higher spatial resolutions (tens of meters, of relevance for e.g., in seismology applications). Note that even though sensitivities of 3.84 pε/√Hz have been demonstrated with an OPC-based scheme [57], these relied on the assistance of ultra-week FBGs which turn the system into a quasi-distributed sensor, impractical for applications over tens to hundred km (due to cost/complexity and losses).

Regarding the sensing range, several ΦOTDR configurations have claimed operation in up to and above 100 km [27,28,30,31,42], but in said works it was not clear what was the strain sensitivity along the fibre. In this work, with the use of distributed amplification and taking advantage of the CP-ΦOTDR high tolerance towards optical noise (allowing operation with trace SNRs of only a few dB), measurements in up to 100 km are demonstrated with strain ASD noise floors of tens of pε/√Hz (strain standard deviations of ≈nε, see Equation (13)) along the entire fibre.

## 7. Applications

The main applications for CP-ΦOTDR involve those of interest for distributed acoustic sensing, such as pipeline protection [59], borehole monitoring [60] and train tracking [61], seismology [62,63] among others. By comparison to other systems, the demodulation of a linear signal with constant (fading free) SNR across sensing channels along the fibre allows for a better starting point for pattern/threat recognition algorithms [59], but also allows for a more efficient use of 2D image processing algorithms, with important applications in areas such as seismology [63].

On the other hand, given the sensor high sensitivity, the CP-ΦOTDR opens the door for a wide range of niche applications, related to distributed sensing with high sensitivity of refractive-index (e.g., chemical sensing) and temperature-based transducers. While several experiments can be envisaged, a list of a few examples already realized in CP-ΦOTDR is listed below.

In temperature-based transducers, experiments on a long range hot-wire anemometer [64], distributed (and discriminatory) mapping of gas [65] and distributed bolometer have been demonstrated [66]. In hot wire anemometry, the measurement of wind speed was demonstrated by applying heat cycles via electrodes (embedded in the fiber coating) and monitoring the amplitude of said temperature cycles. Regarding the gas mapping, it was achieved by monitoring local temperature variations associated with laser emission cycles at certain absorption gas wavelengths (i.e., thus measuring concentration and discriminating gas type). As for the distributed bolometer (allowing for distributed measurement of radiation), it was achieved by monitoring temperature variations of two fibre with coatings with different absorption coefficients.

Regarding experiments monitoring refractive index variations, chemical sensing has been demonstrated. In particular, the diffusion (absorption and desorption) of hydrogen and deuterium into the silica glass of a SMF in a high-pressure steel vessel which was monitored over several weeks [47]. The quadratic electro-optic Kerr effect has also been measured [67] showing, as expected, a refractive index variation proportional to the square of the applied voltage, for ranges between 0–500 V, applied via electrode holes placed symmetrically into the fibre silica matrix.

## 8. Conclusions

In this paper the technology of chirped-pulse (CP-)ΦOTDR is reviewed, ranging from the basic theoretical concepts and principle of operation (Section 2), to optical signal considerations (Section 3), and signal processing and theoretical strain signal dependencies/limits (Section 4).

Early demonstrations of the CP-ΦOTDR used high-performance laboratory equipment (e.g., 40 GS/s digital sampling), which was not truly required, but made for easier conceptual demonstrations. However, by making use of numerous recently published improvements, performances close to the theoretical CRLB limits are currently attainable in certain operation ranges (mainly, metric spatial resolutions, for a given optical SNR) even with low-cost setups using direct detection. In this work, using 1 GS/s digital sampling and a 1 MHz linewidth laser, sensing ranges of up to 100 km are demonstrated (with the use of distributed amplification) with ≈tens-hundreds of pε/√Hz (and down to 15 pε/√Hz for shorter fibres).

While CP-ΦOTDR has demonstrated, to the best of our knowledge, the highest sensitivity in distributed fibre strain measurements without FBG-based assistance (with 3.4 pε/√Hz over 10 km of conventional SMF), further research is required to improve the performance of this relatively recent technology. The theoretical CRLB limits depend on the optical SNR and therefore more advanced pulse modulation schemes/optical configurations and efficient post-processing techniques would allow for an increase of the sensor sensitivity/sensing range. Techniques to efficiently allow for sub-meter spatial resolutions would also be of relevance to further improve the range of applicability of the CP-ΦOTDR.

Additionally, CP-ΦOTDR (and ΦOTDR in general) have traditionally been optimized for acoustic frequencies, aimed at the mainstream DAS applications. However, characterization and optimization for operation at low frequency (<1 Hz) and long-term stability (>24 h) would open the door, or help consolidating, the use of CP-ΦOTDR for non-mainstream DAS applications such as seismology, chemical sensing, temperature-based transducers or fibre characterization.
